# Predictors of Post-Intensive Care Syndrome in ICU Survivors After Discharge: An Observational Study

**DOI:** 10.3390/jcm14176043

**Published:** 2025-08-26

**Authors:** Francesco Gravante, Paolo Iovino, Francesca Trotta, Beatrice Meucci, Marco Abagnale, Stefano Bambi, Gianluca Pucciarelli

**Affiliations:** 1 Department of Biomedicine and Prevention, University of Rome Tor Vergata, 00133 Rome, Italy; francesca.trotta.91@gmail.com (F.T.); meucci.beatrice@gmail.com (B.M.); abagnale.marco@gmail.com (M.A.); gianluca.pucciarelli@uniroma2.it (G.P.); 2 Department of Health Sciences, University of Florence, 50131 Florence, Italy; paolo.iovino@unifi.it (P.I.); stefano.bambi@unifi.it (S.B.)

**Keywords:** post-intensive care syndrome, survivors, predictors, intensive care units, PREDELIRIC score

## Abstract

**Background/Objectives**: Post-intensive care syndrome (PICS) includes new or worsening physical, cognitive, and mental impairments following intensive care unit (ICU) admission. However, its predictors remain poorly defined. This study aimed to identify the predictors of PICS among ICU survivors 30 days after discharge. **Methods**: This prospective, monocentric, observational study was conducted from September 2023 to March 2024. Adult ICU survivors were assessed using the Healthy Ageing Brain Care Monitor to evaluate their physical, cognitive, and mental dimensions. The predictors included age, sex, coma, sedation, clinical severity (APACHE score), risk of ICU delirium (PREDELIRIC score), infection, hospital length of stay, and mechanical ventilation duration. Multivariate linear regression was used to identify independent predictors (*p* < 0.05). **Results**: A total of 90 ICU survivors were enrolled in the study. Higher clinical severity (B = 0.17, *p* = 0.001) and high delirium risk (PREDELIRIC score: B = 3.11, *p* = 0.007) were associated with worse cognitive PICS. Functional PICS was predicted by clinical severity (B = 0.36, *p* = 0.002) and moderate delirium risk (PREDELIRIC score: B = 7.12, *p* = 0.009). Behavioural PICS was inversely associated with coma (B = −6.74, *p* = 0.023) but positively associated with sedation (B = 7.64, *p* = 0.013) and moderate delirium risk (B = 2.24, *p* = 0.031). **Conclusions**: Clinical severity, PREDELIRIC score, sedation, and coma were significant predictors of PICS subdomains. Multidisciplinary teams may be more effective by prioritising targeted screening to identify ICU survivors at elevated risk for PICS using validated predictors such as clinical severity and the PREDELIRIC score, and delivering focused interventions to those most likely to benefit.

## 1. Introduction

In recent years, the development of organ and system support and the quality of care delivered to patients in care settings have grown exponentially [1]. Intensive Care Units (ICUs) deliver highly complex interventions that have significantly increased survival rates in recent years [2]. Despite this, ICU settings expose critically ill patients to different levels of stress during and after discharge [1,3]. ICU survivors experience sequelae attributable to post-intensive care syndrome (PICS), which can last for years.

PICS is a set of post-ICU sequelae that involve patients’ physical, cognitive, and mental dimensions [4]. The physical dimension is related to symptoms such as obvious disability, dyspnoea, weakness, impaired mobility, malnutrition, and sleep disturbance [5]. Cognitive impairment is a set of symptoms attributable to memory loss, dementia, and impaired executive function [5]. Symptoms such as depression, anxiety, posttraumatic stress disorder (PTSD), self-harm, and suicide can be attributed to the mental dimension of the syndrome [3,5]. The social impact on ICU survivors has recently been described as difficult interpersonal relationships, social isolation, employment difficulties, and financial problems [5,6].

Recent studies have highlighted that 95.7% of ICU survivors show at least one PICS criterion [7], whereas 56% show two or more PICS impairments [8]. PICS is a syndrome with a multifactorial aetiology [5,9]. Risk factors for PICS can be classified as non-modifiable (i.e., age, frailty, female sex, disability, pre-existing cognitive impairment, pre-existing mental disorder, and sepsis) [5,9], potentially modifiable (i.e., mechanical ventilation, ICU length of stay, shock, hypoxia, and multiple organ failure) [5], and attributable to ICU admissions (i.e., sedation use, onset of ICU delirium, immobility, sleep disturbance, hyperglycaemia, and negative memories of the ICU) [5,9].

PICS has been associated with adverse outcomes after ICU discharge, such as mortality and healthcare costs [10,11]. A recent longitudinal study reported a prevalence of deaths of 11% during hospitalisation and a 52% mortality rate at two years. The frequencies of PICS at 3, 6, and 12 months were 70%, 60%, and 35%, respectively. Two-year survival was lower in the PICS group (54%) than in the non-PICS group (94%) [11]. In the literature, chronic conditions among ICU survivors have been reported to significantly increase healthcare costs, with mean daily expenses of €8.9 before and €15.4 after ICU admission, thereby highlighting a progressive rise in healthcare expenditures for ICU survivors over the years [12].

Delirium is strongly associated with PICS, as it often leads to cognitive impairment and long-term mental health issues in ICU survivors [7]. Delirium, particularly when prolonged, increases the risk of developing PICS, including physical, cognitive, and mental symptoms, which contribute to worse overall outcomes after ICU discharge. The Prediction of Delirium in ICU patients (PREDELIRIC) score, which predicts delirium risk early in ICU admission, may help identify patients at high risk of delirium and subsequent PICS [13]. It is crucial to identify high-risk groups, detect PICS early, and determine its predictors in ICU survivors, as no study has focused on preventing PICS in this population. Although several studies have been conducted to assess PICS, there remains a lack of consensus on its predictors [2,7,8,14], such as the presence of coma, sedation, APACHE II score, infection, length of stay, and duration of mechanical ventilation. Although there is a growing body of research on the clinical outcomes of critical illnesses, including the physical recovery process, less attention has been devoted to understanding the psychological, cognitive, and social dimensions of recovery [8,15,16]. More specifically, there is a lack of comprehensive knowledge regarding the factors contributing to the development of PICS and the unique needs of ICU survivors during their recovery.

Although several studies have established the association between ICU delirium and the development of PICS and explored its underlying mechanisms [7,15], the predictive approach has primarily focused on identifying patients at high risk of delirium [13]. However, there is a lack of studies regarding the ability of the PREDELIRIC score, applied within the first 24 h of ICU admission, to predict the subsequent onset of PICS after discharge.

The knowledge gap surrounding PICS arises from the complexity of post-ICU recovery, as multiple factors, such as illness severity, duration of ICU stay, and pre-existing comorbidities, may influence the development of PICS [9,17]. Despite evidence that ICU survivors often experience long-term impairments across multiple domains, predicting PICS remains difficult because of the complex relationship between its dimensions [18,19]. This lack of understanding limits healthcare professionals’ ability to effectively support survivors and tailor interventions to alleviate the burden of PICS. Moreover, this study provides important insights into the identification of high-risk individuals for post-ICU sequelae.

### Aim

This study aimed to identify intensive care–related predictors of PICS in ICU survivors following ICU discharge.

## 2. Materials and Methods

### 2.1. Study Design and Research Questions

We conducted a monocentric prospective observational study in the ICU of a university hospital in Italy. This research report was written according to the Strengthening the Reporting of Observational Studies in Epidemiology (STROBE) guidelines. The research question that guided our study was as follows: “Which predictors contribute to the onset of post-intensive care syndrome in ICU survivors after discharge?”

### 2.2. Data Collection and Participants

**Figure 1:**
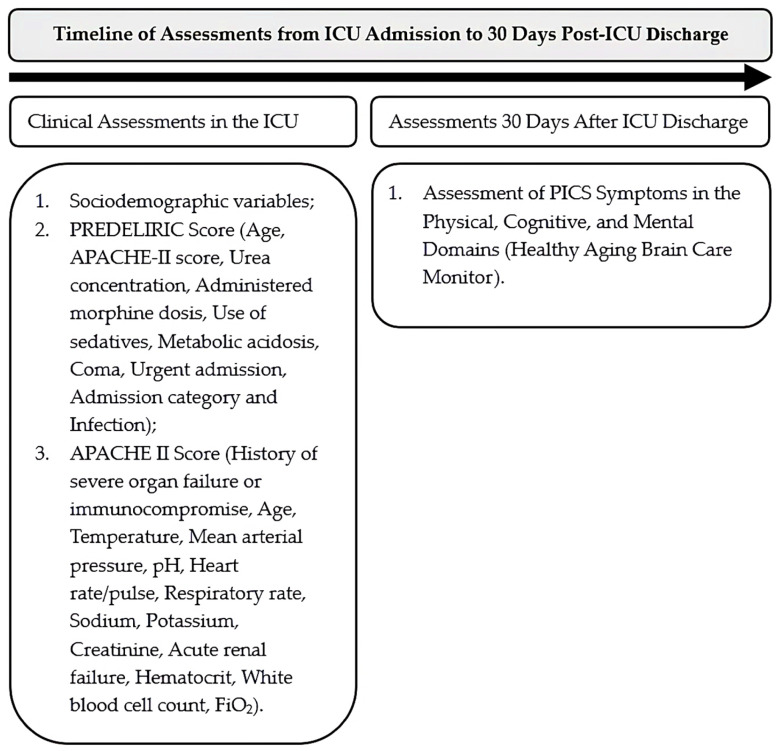
Timeline for assessment pre- and post-ICU.

This study was conducted in two phases. In the first phase, information on variables and predictors (Section 2.3) was collected, including assessments of the PREDELIRIC and APACHE II scores. Subsequently, one month after discharge from the ICU, patients were assessed for PICS symptoms using the Healthy Aging Brain Care Monitor (Figure 1).

All consecutive adult survivors discharged from the ICU between 1 September 2023 and 1 March 2024 were enrolled in the study and assessed one month after ICU discharge. ICU survivors were discharged from a surgical ICU that served postoperative patients after liver transplantation and critically ill patients with medical diagnoses. One month after ICU discharge, survivors who provided written informed consent were contacted via telephone and enrolled in the study. An ICU nurse trained in PICS evaluation collected variables of interest during hospitalisation, contacted ICU survivors, and administered the PICS assessment score (Healthy Aging Brain Care Monitor) to evaluate PICS’s physical, cognitive, and mental dimensions one month after ICU discharge. The inclusion criteria for this study were ICU survivors over 18 years of age, with preserved language functions at discharge, who received mechanical ventilation, and remained in the ICU for at least 48 h.

The following categories of patients were excluded: survivors discharged from the ICU for palliative care or transferred to hospice; certified diagnosis of delirium and/or dementia before admission to the ICU; brain injuries; not speaking or understanding Italian; sensory injuries (e.g., visual or hearing loss); and survivors not discharged home at the moment of the call for recruitment.

### 2.3. Variables and Predictors

Sociodemographic variables such as sex, age, ethnicity, formal education, smoking, and alcohol habits were collected from electronic medical charts. The factors associated with PICS and its subdomains were derived from a literature review of theoretical and empirical studies [9]. The target intensive care–related predictors included the presence of coma, sedation, clinical severity, PREDELIRIC score, infection, length of hospital stays, and duration of mechanical ventilation.

### 2.4. Outcome

The primary outcome of this study was the development of PICS after ICU discharge.

### 2.5. Instruments

The Healthy Aging Brain Care Monitor (HABCM) was used to measure the risk of developing delirium after discharge [20]. This self-report tool comprises three dimensions (cognitive, functional, and behavioural) and 27 items. Patients indicated how often they experienced specific delirium-related symptoms in the preceding two weeks. Subscales include the cognitive dimension (six items), functional dimension (11 items), and behavioral dimension (10 items). Each item has a four-point Likert scale answer option: 0 points for “Not at all” (0–1 day), 1 point for “several days” (2–6 days), 2 points for “more than half the days” (7–11 days), and 3 points “almost daily” (12–14 days). The total score on the HABC-M SR ranges from 0 to 81, the cognitive score ranges from 0 to 18, the functional score ranges from 0 to 33, and the behavioural score ranges from 0 to 30. HABC-M SR score yields four risk levels: normal, mild, moderate, and severe. The scoring is as follows: HABC-M SR total score (normal ≤ 14, mild = 15–23, moderate = 24–35, severe ≥ 36), cognitive dimensions (normal ≤ 4, mild = 5–8, moderate = 9–11, severe ≥ 12), functional dimensions (normal ≤ 3, mild = 4–6, moderate = 7–11, severe ≥ 12), and psychological dimensions (normal ≤ 5, mild = 6–7, moderate = 8–11, severe ≥ 12). The internal consistency of the HABC-M SR was satisfactory in this study, with Omega coefficients of 0.81 for the cognitive dimension, 0.96 for the functional dimension, 0.71 for the behavioural dimension, and 0.94 for the total scale.

The PREDELIRIC score is a reliable predictor of ICU delirium in critically ill patients. Ten risk factors (age, APACHE II score, urea concentration, administered morphine dose, use of sedatives, metabolic acidosis, coma, urgent admission, admission category, and infection) collected in the first 24 h after admission combined yielded a risk percentage as follows: low (score < 20%), moderate (score between 21–40%), high (score between 41–60%), and very high (score > 60%) [21]. Its psychometric properties include high sensitivity (91.3%) and specificity (64.4%), making it a reliable tool for identifying patients at risk of delirium [13].

The APACHE II score is used to assess the severity of illness in critically ill patients. It includes 12 physiological parameters (history of severe organ failure or immunocompromise, age, temperature, mean arterial pressure, pH, heart rate/pulse, respiratory rate, sodium, potassium, creatinine, acute renal failure, haematocrit, white blood cell count, FiO_2_) and chronic risk factors such as age and pre-existing conditions. A score above 20 is generally associated with a higher risk of mortality [22].

### 2.6. Statistical Analysis

Sociodemographic and clinical characteristics were described using summary statistics (mean, standard deviation, absolute frequency, and percentage). Four multivariable linear regression models using the least squares method were fitted to understand the predictors associated with the HABCM total score and the cognitive, functional, and behavioural scores. The parameters are reported as unstandardised coefficients (B) and standard errors (SE). The determination coefficient (R2) for each model is reported. The sample size was calculated based on an expected PICS symptom prevalence of 13% (*p* = 0.13) [23], with a desired absolute precision of 7% (d = 0.07) and a 95% confidence level [24]. This yielded a minimum required sample size of approximately 89 participants per group. All statistical tests were two-sided and considered statistically significant at *p* < 0.05. SPSS^®^ v.25 and STATA^®^ MP 14 were used to compute descriptive statistics and estimate regression models, respectively.

### 2.7. Ethical Considerations

This study was approved by the Ethics Committee of the Antonio Cardarelli Hospital in Naples, Italy (Prot. 00014635–31 May 2023). Written informed consent was obtained from all participants prior to discharge. Privacy was guaranteed in accordance with the research recommendations of the Declaration of Helsinki.

## 3. Results

**Figure 2:**
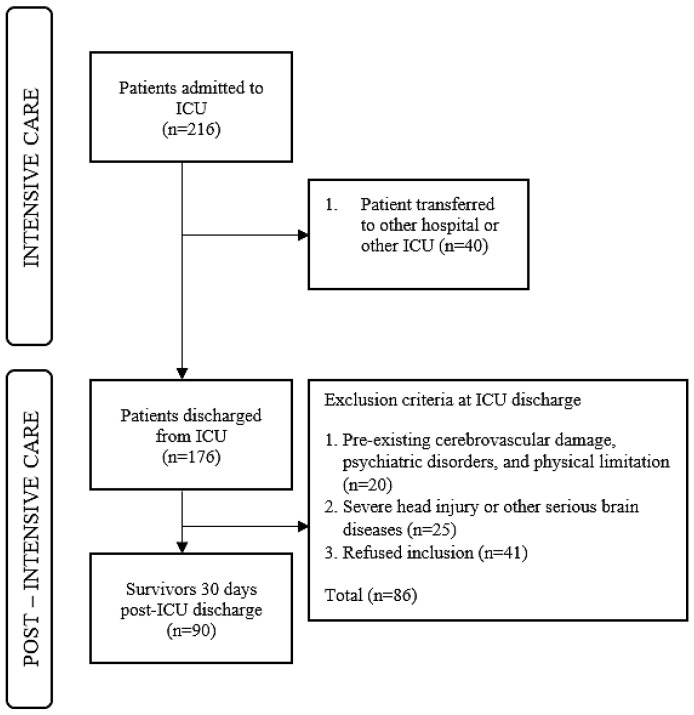
Flow chart of survivors through the study.

During the study period, 216 patients were admitted to the ICU, of whom 90 (41.6%) met the inclusion criteria after discharge. The sample consisted of 90 ICU survivors after discharge; further details are shown in Figure 2.

### 3.1. Sociodemographic Characteristics of the Sample and PICS

Table 1 shows the sociodemographic and clinical characteristics of the participants (N = 90). Briefly, most patients were male (58.9%, n = 53), had an average age of 63.3 years (SD = 16.03), and had a formal education of less than eight years. During the ICU stay (mean days = 16.51, SD = 14.06), most participants were in a comatose state (57.8%), sedated (57.8%), and under mechanical ventilation for an average of 5.61 days (SD = 6.02).

Based on the total HABCM scores, the patients exhibited mild overall symptoms (19.54, SD = 16.08). They also had mild cognitive (5.16, SD = 4.47) and behavioural symptoms (5.14, SD = 4.04) but exhibited moderate symptoms in the functional scale domain (9.24, SD = 10.12). Supplementary Table S1 shows the frequency of responses to the individual items of the HABCM. The answers to these items indicated a general predominance of situations, signs, and symptoms that were never or sometimes experienced. However, patients reported frequent problems with their safety (item 15), interest or pleasure in doing things, hobbies, or activities (item 18), and feeling down, hopeless, or depressed.

### 3.2. Multivariate Regression Models

Table 2 presents the results of the multivariable regression models. In the first model, those with a high risk of developing delirium (B = 12.45, *p* = 0.004) and those with a higher disease severity (B = 0.60, *p* = 0.002) were more likely to develop PICS after ICU discharge than their counterparts.

In the second model, those with a high risk of delirium (B = 3.11, *p* = 0.007) and those with high disease severity (B = 0.17, *p* = 0.001) were more likely to exhibit cognitive impairment than others. Similarly, in the third model, those exhibiting worse clinical severity (B = 0.36, *p* = 0.002) and a moderate risk of delirium (B = 7.12, *p* = 0.009) predicted poorer functional impairment. Finally, those who were in a coma during the ICU stay had a lower risk of behavioural PICS than the other group (B = −6.74, *p* < 0.023), while sedation (B = 7.64, *p* = 0.013) and moderate risk of delirium (B = 2.24, *p* = 0.031).

## 4. Discussion

This study aimed to identify the predictors of PICS onset in ICU survivors after discharge. The results indicated that clinical severity and risk of delirium were significant predictors of the onset of PICS (HABC-M SR total score), particularly in the cognitive and functional domains. Additionally, sedation, coma, and risk of delirium were independent predictors of psychological deficits in survivors after ICU discharge.

Consistent with the literature, several diseases included in the APACHE model have been identified as independent predictors of PICS in ICU survivors, including sepsis, respiratory failure, and acute renal failure [11]. These conditions exacerbate critical illness and significantly affect recovery outcomes. For example, sepsis is associated with widespread inflammation and multiorgan dysfunction, both of which can increase the risk of PICS [25]. Respiratory failure, which often requires prolonged mechanical ventilation, can lead to muscle weakness and cognitive impairment, further complicating the recovery process [26]. Similarly, acute renal failure may result in electrolyte imbalance and metabolic disturbances that contribute to physical and cognitive dysfunction [27]. These findings emphasise the need for early identification and management of these critical conditions to mitigate their long-term effects on ICU survivors.

Several clinical factors, including electrolyte imbalances such as hypocalcaemia, hypokalaemia, hypophosphataemia, hypomagnesaemia, hypermagnesemia, and acute hypercapnia, worsen muscle weakness [28]. For instance, hospitalised patients who experience an increase in carbon dioxide tension (Pco2) may enter a vicious cycle in which elevated Pco2 levels further reduce muscle strength [29]. Many of these variables are recognised as risk factors within the APACHE II model, which assesses the severity of illness and predicts outcomes in critically ill patients [30]. These clinical factors likely exacerbate the physical impairments observed in ICU survivors, potentially leading to long-term muscle weakness and functional decline after discharge [31]. These findings underscore the importance of monitoring and managing physiological disturbances to minimise their impact on recovery and guide clinical decisions in the ICU. Our findings align with these results, as compromised clinical conditions significantly influence ICU outcomes, particularly in vital organs essential for survival following intensive treatment [1]. The APACHE score, an established mortality index, evaluates vital functions that are often compromised upon ICU admission. The severity of critical illness negatively impacts PICS and contributes to ICU readmission rates of 22–27% within 30 days and 33–36% within 90 days, particularly in patients with sepsis [32,33]. Common readmission diagnoses include sepsis, congestive heart failure, pneumonia, acute renal failure, and respiratory failure [34].

Severe physical impairment symptoms were the most prevalent, and clinical severity and risk of delirium were identified as predictors of these impairments. Bed rest and muscle weakness induced by ICU conditions contribute to physical decline [35]. Electrolyte imbalance and hypercapnia exacerbate muscle weakness [28]. Many of these risk factors were included in the APACHE II score.

Coma, sedation, and risk of delirium were associated with the onset of PICS. Induced coma and sedation are routinely used in the ICU to manage the pain and anxiety of patients. Induced coma protects the brain during major neurosurgery, treats refractory status epilepticus, and manages intracranial hypertension after traumatic brain injury [36]. However, deep sedation and prolonged immobility increase the risk of delirium, which is linked to a higher likelihood of developing PICS [37]. This condition was not observed in the present study, as mild analgesia was often used instead, and patients were precociously weaned from sedation. However, they can complicate drug interactions and patient recovery, particularly given their unpredictable pharmacokinetics in critically ill patients [38,39]. This complexity makes it difficult to achieve therapeutic benefits without exacerbating the harm caused by the disease.

Identifying the predictors of PICS has significant implications for clinical practice and the broader scientific community. Understanding these predictors will enable healthcare professionals to identify at-risk ICU survivors more accurately, thereby facilitating early interventions that may prevent or mitigate the long-term effects of PICS [19]. This is particularly important given the multifactorial nature of PICS, which encompasses physical, cognitive, and psychological impairments that can profoundly affect an individual’s quality of life and long-term recovery [15].

The importance of early detection and post-ICU follow-up cannot be overstated for healthcare professionals, especially those in community settings [11]. Multidisciplinary teams, including nurses, physicians, psychologists, and rehabilitation specialists, are crucial for managing PICS. These teams must collaborate to provide comprehensive care that addresses the immediate and long-term needs of ICU survivors [40]. Post-discharge care should address PICS predictors, such as the severity of critical illness, pre-existing conditions, and interventions, such as sedation and mechanical ventilation, all of which contribute to the onset of symptoms [41].

From a scientific perspective, further research on the predictors of PICS will continue to advance our understanding of the pathophysiology of this syndrome, enabling the development of targeted interventions and preventive strategies [40]. These findings highlight the need for standardised screening tools and care protocols to ensure that ICU survivors receive adequate follow-up and support.

These results represent an important first step in planning early rehabilitation and post-ICU rehabilitation. Early identification of risk factors could enable the stratification of high-risk ICU survivors and the implementation of individualised rehabilitation programs, thereby optimising recovery trajectories and improving long-term physical, cognitive, and psychological outcomes [2]. Ultimately, better recognition and management of PICS can significantly improve outcomes for ICU survivors and reduce the burden on the healthcare system.

### Limitations

This study had several limitations. First, the sample size of 90 ICU survivors in the monocentric setting may not fully represent the broader population of ICU patients, limiting the generalisability of the findings. In addition, the ICU analyzed in this study admits only surgical patients with severe liver failure who have undergone transplantation. This is a limitation, as the results may not apply to other types of ICU survivors. For this reason, it would be valuable to extend the analysis to different case mixes. Additionally, the study relied on self-reported data from ICU survivors, which could have introduced recall bias or inaccuracies in the symptom reporting. The cross-sectional design also means that causality between predictors and PICS outcomes cannot be definitively established. Furthermore, the study focused on ICU survivors 30 days post-discharge; however, the long-term trajectory of PICS symptoms and recovery remains unclear, as further follow-up was not conducted [42,43]. Finally, while clinical severity and risk of delirium were identified as significant predictors, other potential factors influencing PICS, such as social support, lifestyle changes, and socioeconomic status, were not considered, which may have provided a more comprehensive understanding of the syndrome predictors.

## 5. Conclusions

This study highlighted the significant role of clinical severity and the PREDELIRIC score in predicting the onset of PICS in ICU survivors. These findings emphasise the need for the early identification of high-risk individuals to facilitate timely intervention. Given the multifaceted nature of PICS, including cognitive, functional, and psychological impairments, a comprehensive multidisciplinary care approach is crucial for effective management. The findings of this study have several implications for clinical practice, particularly in the early identification and management of ICU survivors at risk of PICS. Using validated predictors such as clinical severity and the PREDELIRIC score may guide multidisciplinary teams in implementing tailored monitoring, multicomponent interventions, preventive strategies, and targeted ICU and post-ICU rehabilitation programs. Moreover, prioritising early weaning from sedatives and carefully managing coma conditions may further reduce the risk of adverse cognitive, functional, and behavioural outcomes.

However, given the study’s limitations, further longitudinal research is needed to explore the long-term trajectory of PICS, refine predictive models, and account for additional factors such as social support, lifestyle changes, and socioeconomic status, while also including diverse types of ICU survivors. Such efforts would ultimately contribute to improving post-ICU care and recovery outcomes.

## Figures and Tables

**Table 1:** Sociodemographic and Clinical Characteristics of Participants (n = 90).

Sociodemographic and Clinical Characteristics	N (%) or m (SD)
Gender (female)	37 (41.1)
Age (years)	63.3 (16.03)
Ethnicity (Italian)	90 (100)
Formal education (>8 yrs)	33 (36.7)
Smoking habit	
Use in the past	49 (54.4)
Actual use	5 (5.6)
Alcohol habit (no)	
Never	78 (86.7)
Use in the past	12 (13.3)
Coma (yes)	52 (57.8)
Sedation (yes)	52 (57.8)
APACHE II score	15.33 (9.25)
Predeliric score	
low risk	30 (33.3)
moderate risk	37 (41.1)
high risk	16 (17.8)
very high risk	7 (7.8)
Length of stay (days)	16.51 (14.06)
Mechanical ventilation (days)	5.61 (6.02)
HABCM	
Cognitive score (0–18)	5.16 (4.47)
Functional score (0–33)	9.24 (10.12)
Behavioural score (0–30)	5.14 (4.04)
Total score (0–81)	19.54 (16.08)

Legend. SD, standard deviation; m, mean; n, absolute frequency; HABCM, Healthy Aging Brain Care Monitor; APACHE II, Acute Physiologic Assessment and Chronic Health Evaluation II.

**Table 2:** Multivariable linear regression models of variables associated with PICS scores at discharge (n = 90).

	Model 1			Model 2			Model 3			Model 4		
	Total Score			Cognitive Score			Functional Score			Behavioral Score		
	B	SE	*p*	B	SE	*p*	B	SE	*p*	B	SE	*p*
Gender (female)	−0.41	3.05	0.893	−0.48	0.81	0.556	0.50	1.92	0.794	−0.43	0.85	0.609
Age (one year-unit)	−0.09	0.11	0.389	−0.02	0.03	0.548	−0.08	0.07	0.224	0.01	0.03	0.811
Coma (yes)	−9.26	10.53	0.380	−0.99	2.80	0.724	−1.53	6.61	0.530	−6.74	2.93	0.023
Sedation (yes)	12.71	10.53	0.231	1.07	2.81	0.703	4.18	6.63	0.530	7.46	2.94	0.013
APACHE II score	0.60	0.18	0.002	0.17	0.05	0.001	0.36	0.11	0.002	0.07	0.05	0.158
PREDELIRIC score *												
1	−0.20	3.67	0.957	−1.57	0.98	0.112	−0.87	2.31	0.707	2.24	1.02	0.031
2	12.45	4.21	0.004	3.11	1.12	0.007	7.12	2.65	0.009	2.22	1.17	0.061
Length of stay (days)	−0.13	0.18	0.463	−0.03	0.05	0.554	−0.16	0.11	0.156	0.06	0.05	0.249
Mechanical ventilation (days)	0.38	0.40	0.351	0.12	0.11	0.271	0.36	0.25	0.154	−0.11	0.11	0.344
Intercept	11.36	8.37	0.179	3.49	2.23	0.122	6.37	5.27	0.231	1.51	2.33	0.519
R^2^	0.33			0.37			0.33			0.18		
Adjusted R^2^	0.26			0.31			0.25			0.09		

Legend. B, unstandardized coefficient; SE, standard error; *p*, *p*-value; R^2^, determination coefficient; PICS, post-intensive care syndrome; APACHE II, Acute Physiologic Assessment and Chronic Health Evaluation II, Notes. * Class of PREDELIRIC (1) = low risk/moderate risk vs. (2) = very high/high risk of the onset of ICU delirium in ICU survivors.

## Data Availability

The data supporting this study’s findings are available from the corresponding author [F.G.] upon reasonable request.

## Supplementary Materials

The following supporting information can be downloaded at: https://www.mdpi.com/article/10.3390/jcm14176043/s1, Table S1: Descriptives of HABCM questionnaire items (n = 90).
