# Supplementary material for: Predictors of Post-Intensive Care Syndrome in ICU Survivors After Discharge: An Observational Study
Source: J Clin Med. 2025 Aug 26;14(17):6043. doi: 10.3390/jcm14176043 (PMC12429453; doi:10.3390/jcm14176043)
Supplement: Supplementary file 1 [file jcm-14-06043-s001.zip › jcm-3788205-supplementary.pdf]

Supplementary Table S1. Descriptives of HABCM questionnaire items (n = 90).

Item	Mean (SD)	Not at all (n, %)	Several days (n, %)	More than half of the days (n, %)	Almost daily (n, %)
Over the past 2 weeks, how often did **you** have problems with:					
1. Judgment or decision-making	0.98 (1.25)	51 (56.7)	9 (10)	11 (12.2)	19 (21.1)
2. Repeating the same things over and over, such as questions or stories	0.59 (0.87)	56 (62.2)	19 (21.1)	11 (12.2)	4 (4.4)
3. Forgetting the correct month or year	0.57 (0.81)	53 (58.9)	27 (30)	6 (6.7)	4 (4.4)
4. Handling complicated financial affairs such as balancing checkbook, income taxes, and paying bills	0.89 (1.24)	56 (62.2)	6 (6.7)	10 (11.1)	18 (20)
5. Remembering appointments	1.23 (1.24)	38 (42.2)	15 (16.7)	15 (16.7)	22 (24.4)
6. Thinking or memory	0.90 (1.23)	53 (58.9)	12 (13.3)	6 (6.7)	19 (21.1)
7. Learning how to use a tool, appliance, or gadget	0.87 (1.16)	53 (58.9)	10 (11.1)	13 (14.4)	14 (15.6)
8. Planning, preparing, or serving meals	0.89 (1.23)	54 (60)	11 (12.2)	6 (6.7)	19 (21.1)
9. Taking medications in the right dose at the right time	0.93 (1.23)	51 (56.7)	13 (14.4)	7 (7.8)	19 (21.1)
10. Walking or physical ambulation	0.92 (1.25)	53 (58.9)	11 (12.2)	6 (6.7)	20 (22.2)
11. Bathing	0.93 (1.20)	49 (54.4)	16 (17.8)	7 (7.8)	18 (20)
12. Shopping for personal items like groceries	0.87 (1.12)	48 (53.3)	21 (23.3)	6 (6.7)	15 (16.7)
13. Housework or household chores	0.73 (1.03)	51 (56.7)	23 (25.6)	5 (5.6)	11 (12.2)
14. Being left alone	0.88 (1.09)	46 (51.1)	22 (24.4)	9 (10)	13 (14.4)
15. Your safety	1.10 (1.18)	40 (44.4)	19 (21.1)	13 (14.4)	18 (20)
16. Your quality of life	0.68 (1.06)	59 (65.6)	11 (12.2)	10 (11.1)	10 (11.1)
17. Falling or tripping	0.44 (0.75)	61 (67.8)	21 (23.3)	5 (5.6)	3 (3.3)
18. Less interest or pleasure in doing things, hobbies or activities	0.80 (0.85)	40 (44.4)	31 (34.4)	16 (17.8)	3 (3.3)
19. Feeling down, depressed, or hopeless	0.77 (0.90)	42 (46.7)	34 (37.8)	7 (7.8)	7 (7.8)

20. Resisting help from others or getting agitated	0.73 (0.98)	50 (55.6)	22 (24.4)	10 (11.1)	8 (8.9)
21. Feeling anxious, nervous, tense, fearful, or panicky	0.43 (0.77)	63 (70)	18 (20)	6 (6.7)	3 (3.3)
22. Believing others are stealing from you or planning to harm you	0.29 (0.72)	75 (83.3)	9 (10)	2 (2.2)	4 (4.4)
23. Hearing voices, seeing things, or talking to people who are not there	0.23 (0.60)	76 (84.4)	8 (8.9)	5 (5.6)	1 (1.1)
24. Poor appetite or overeating	0.50 (0.82)	60 (66.7)	19 (21.1)	7 (7.8)	4 (4.4)
25. Falling asleep, staying asleep, or sleeping too much	0.32 (0.65)	68 (75.6)	17 (18.9)	3 (3.3)	2 (2.2)
26. Acting impulsively, without thinking through the consequences of your actions	0.33 (0.62)	66 (73.3)	19 (21.1)	4 (4.4)	1 (1.1)
27. Wandering, pacing, or doing things repeatedly	0.74 (0.96)	48 (53.3)	24 (26.7)	11 (12.2)	7 (7.8)

Legend. SD, standard deviation; n, absolute frequency; HABCM, Healthy Aging Brain Care Monitor.
